# Non invasive blood glucose estimation using green light photoplethysmography and machine learning

**DOI:** 10.3389/fdgth.2026.1705086

**Published:** 2026-02-17

**Authors:** Khadija Khan, Laraib Malik, Abdul Qadeer Khan, Saadullah Farooq Abbasi, Theodoros N. Arvanitis

**Affiliations:** 1 Department of Biomedical Engineering, Riphah International University, Islamabad, Pakistan; 2 Department of Electronic, Electrical and Systems Engineering, University of Birmingham, Birmingham, United Kingdom

**Keywords:** machine learning, non-invasive glucose monitoring, optical technology, photoplethysmography signals, signal processing

## Abstract

Non-communicable diseases, such as diabetes, are the leading cause of mortality worldwide. Effective diabetes management is crucial for ensuring the well-being of diabetics. Existing glucose monitoring technologies are often invasive and uncomfortable, eliciting anxiety among patients. Non-invasive procedures offer a promising solution for these issues, but their widespread adoption is restricted by cost and accuracy constraints. This study investigates the use of green light Photoplethysmography (PPG) signals for non-invasive blood glucose monitoring. A custom-designed PPG acquisition setup was developed to collect PPG data from 80 subjects under controlled conditions. Simultaneously, reference capillary blood glucose readings were obtained using a lancing device to serve as the gold standard. Signals were enhanced by applying different processing techniques and 32 features were extracted, which were scaled and subjected to correlation analysis to retain the highly correlated features. Feature engineering further optimized the feature set, which was then used to train and validate regression models. Model performance was evaluated using R^2^ (coefficient of determination), mean absolute error (MAE), and bias analysis across glucose ranges. Among the tested models, the Random Forest Regression (RFR) showed the best performance with an R^2^ value of 0.92 and MAE of 4.8 mg/dL. Predicted glucose levels demonstrated minimal bias across glucose ranges, with mean differences of −5.11 ± 0.80 mg/dL (<90 mg/dL), −3.68 ± 1.24 mg/dL (90–120 mg/dL), and −5.61 ± 0.75 mg/dL (≥120 mg/dL). The findings demonstrate that PPG signals in the green light spectrum effectively reflect blood glucose levels, supporting their potential for non-invasive glucose monitoring.

## 1 Introduction

Diabetes is one of the most common public health long-term condition worldwide. According to the International Diabetes Federation's 2022 forecast, diabetes affects one out of every ten people worldwide (1). Diabetes is becoming increasingly common, particularly in developing nations (2). This widely spread disease requires careful and constant monitoring as it leads to several chronic health issues, if left neglected (2, 3). Regular glucose checks for people with diabetes are crucial for monitoring and controlling the disease. To self-monitor the glucose level on a daily basis, the finger pricking method is used which contributes to patient anxiety and discomfort (4). Considering the risk of potential infections and associated anxiety due to pain, there is a gap for a painless but clinically approved and effective method of measuring glucose level. To address these concerns, Continuous Glucose Monitoring (CGM) systems have become the gold standard for individuals on insulin therapy. These systems allow for painless and continuous monitoring by measuring interstitial glucose levels and transmitting real-time data to smartphone applications every minute for extended periods, depending on the system (5). CGMs have currently drawn attention for use among non-diabetic groups as well e.g., those with metabolic syndrome, athletes, and individuals who prioritize well-being, for tracking glycemic patterns and healthy behavioral changes (6). However, despite these advancements, achieving a truly non-invasive and equally accurate alternative remains a challenge, as current non-invasive methods struggle to match the accuracy of invasive approaches (7).

PPG uses optical sensors to capture changes in blood volume by shining light onto the skin and analyzing how much light is absorbed or reflected off blood vessels. Since glucose content in blood impacts variables like transparency of tissues, vascular tone, and local hemodynamics, it can impact PPG signal traits as well (8). For instance, high glucose levels can modify endothelial function, elevate blood viscosity, and alter stiffness in arteries; all of which subtly modify the shape and timing of the PPG waveform. With analysis of parameters like amplitude, rising time, and pulse width through complex algorithms, PPG signals are therefore adequately utilized for non-invasive estimation of glucose level in blood without any pain or direct contact with bodily fluids (9). A standard PPG device includes both a light source and a photodetector. It operates by illuminating the skin and measuring the fluctuations in reflected light caused by the pulsatile flow of blood (9, 10).

With the substantial advancement in the design and development of non-invasive wearable technologies, wearable PPG sensors have become popular for monitoring heart rate (HR) because of their user-friendliness, low cost, and ease of use (11). Beyond usability, recent studies show that wrist/finger PPG in consumer wearables provides accurate resting HR, with errors typically below 3% at rest but increasing during vigorous movement. Similarly, heart rate variability (HRV) derived from PPG aligns closely with ECG at rest, though agreement decreases under stress or high activity. Arrhythmia screening via smartwatch-based PPG demonstrates high diagnostic accuracy, while SpO₂ measurements show small average bias but wider limits of agreement (12). A recent umbrella review further confirmed these trends, reporting pooled Atrial Fibrillation detection sensitivity and specificity of 100% and 95%, mean HR bias within ±3%, and mean absolute SpO₂ differences of approximately 2% (13).

Extending beyond cardiovascular monitoring, newer reviews highlight the broader clinical reliability of PPG-based wearables, with diagnostic accuracies exceeding 90% for cardiovascular and sleep disorder assessments (14). In metabolic monitoring, optimized near-infrared wavelengths combined with deep learning models achieved mean absolute relative errors (MARE) of 8%–12%, with over 90% of predictions within Clarke Error Grid Zones A and B (15). Integrating PPG with complementary sensor modalities such as accelerometer and temperature has further improved metabolic estimation accuracy, reaching up to 87% under controlled conditions in large-scale evaluations involving over 25,000 participants. Collectively, these findings establish the growing precision and clinical credibility of PPG-based wearables for cardiovascular monitoring and broader health assessment. Beyond cardiovascular applications, the hemodynamic insights obtained from PPG waveforms can be extended toward metabolic analysis, particularly for non-invasive estimation of Blood Glucose Level (BGL) in the body (9, 10, 16).

Out of all the existing techniques, the cost- effective, simplest, and commonly used method for blood glucose monitoring is near infrared spectroscopy (NIR) along with PPG (17). However, PPG is sensitive to bodily movements. Artifacts from body movement affect the variations in blood volume at the measurement site, distorting the true PPG waveform (18). PPG signals typically have a frequency range of 0.5–5 Hz, whereas motion artifacts span from 0.01 to 10 Hz.

In context of obtaining PPG signal by using green light, its shorter wavelength allows penetration into the shallow portions of relatively hard tissues, and the sensor's connection to the skin improves measurement reliability. However, green light penetrates the skin at a shallower depth than red (approximately 660 nm) and near-infrared (NIR) light, making it more sensitive to motion artifacts, as even minor movements can affect readings (19). Moreover, existing literature indicates that melanin absorption can affect how green light penetrates through different skin types. This variability in the absorption might impact the signal strength and accuracy in glucose monitoring (20). In addition to melanin-related optical effects, motion, contact pressure, ambient light, peripheral perfusion/temperature, and sensor placement are established sources of variability in PPG signals and wearable accuracy, as summarized in recent reviews (12, 13). Although green light has a shorter wavelength and is less penetrative in dense tissues such as muscles and bone, it still penetrates sufficiently to detect blood pulsations and is less affected by the DC component of tissues (21). Given these complexities, and the fact that the PPG signal is quasiperiodic and non-stationary, advanced signal processing techniques are required to ensure accurate measurements (22).

In the context of non-invasive glucose monitoring using PPG signals, two common categories of features are peak-based features and statistical features extracted for analysis. Statistical features explain the variability and distribution of PPG signals (23). However peak features provide insights into the dynamic changes in volume of blood and pattern of arterial perfusion related to the cardiac cycle (24). Accurate results of glucose level in blood from physiological signals is a key challenge for non-invasive monitoring. To address this challenge, various Machine Learning (ML) techniques have been investigated, including regression and classification (25). The aim of such regression models is to set up a mathematical relationship between glucose level and corresponding input features.

## 2 Relevant work

The techniques employed in previous studies for non-invasive blood glucose estimation (26–30), along with their limitations, are summarized in Table 1.

**Table 1:** Related studies existing in the literature with the notable contribution in non-invasive glucose monitoring.

Research Work	Year	Technique used	Limitations
Chen et al. (31)	2024	PPG (Wavelength not specified)	Server-based processing; not validated for different environments
Susana et al. (32)	2023	PPG using Near Infrared LEDs	Limited reliability under variable physiological conditions
Anupongongarch et al. (28)	2022	PPG using Near infrared and Red light	Motion artifacts and environmental change affect signal quality
Zhang et al. (26)	2020	Smartphone PPG acquired through camera videos	Lower precision due to reliance on smartphone camera quality and lighting
Li et al. (33)	2019	Polarized Light	High sensitivity to skin pigmentation and interference from ambient light

Building on the mentioned techniques, Gupta et al. (9) worked on non-invasive blood glucose measurement technique by combining transmission and reflecting data acquisition technologies with red and green LEDs. Random Forest (RF) regression was used to estimate the glucose level. Nampoothiri et al. (16) developed a PPG-based wearable device utilizing Red and Infrared (IR) wavelengths for non-invasive blood glucose monitoring. Their study primarily focused on signal acquisition and statistical feature extraction without integrating advanced signal processing techniques or ML models. These limitations highlight the need for further investigation to establish a definitive relationship between specific wavelengths and glucose estimation. Al-dhaheri et al. (34) proposed a low-cost non-invasive system utilizing NIR spectroscopy and PPG. An ML Linear regression model was applied to get BGL but multiple regression analysis for further improvement is required. Furthermore, Pal et al. (25) introduced the application of an ML algorithm for pre-processing the data, which further enhanced the accuracy of glucose measurements. The BGL of all subjects was tested using patterns obtained from uncovered fiber under an AC magnetic field, which surpassed the performance of other tested configurations. In addition to these technical approaches, Alhmiedat et al. (35) developed the SARA robot system to assist children in diabetes self-management using interactive learning, entertainment, and monitoring. The system uses a humanoid robot to lead children in daily routines and blood sugar monitoring, feeding data back to healthcare providers through a cloud-based platform. However, the system uses traditional blood glucose monitoring, integration with non-invasive technologies like green light-based PPG in such platforms would greatly improve comfort, eliminate needle anxiety, and promote long-term compliance, especially in pediatric practice.

Considering the discussed limitations and the ongoing need for accurate, painless, and cost-effective approaches, this study explores the potential of green light-based PPG signals for blood glucose monitoring. PPG signals were collected from the fingertip using a specialized pulse sensor with a green LED source, while reference capillary blood glucose readings were obtained using a lancing device, for validation. To enhance signal quality, advanced signal processing techniques were applied for proper filtration and extraction of relevant features before employing ML models for glucose prediction. By comparing non-invasive and finger-prick data, this research aims to assess the feasibility of PPG-based glucose monitoring as a reliable alternative to traditional methods. Furthermore, the study evaluates multiple ML models to determine their efficacy for practical implementation, contributing to the development of accessible and accurate non-invasive glucose monitoring solution.

## 3 Methodology

**Figure 1:**
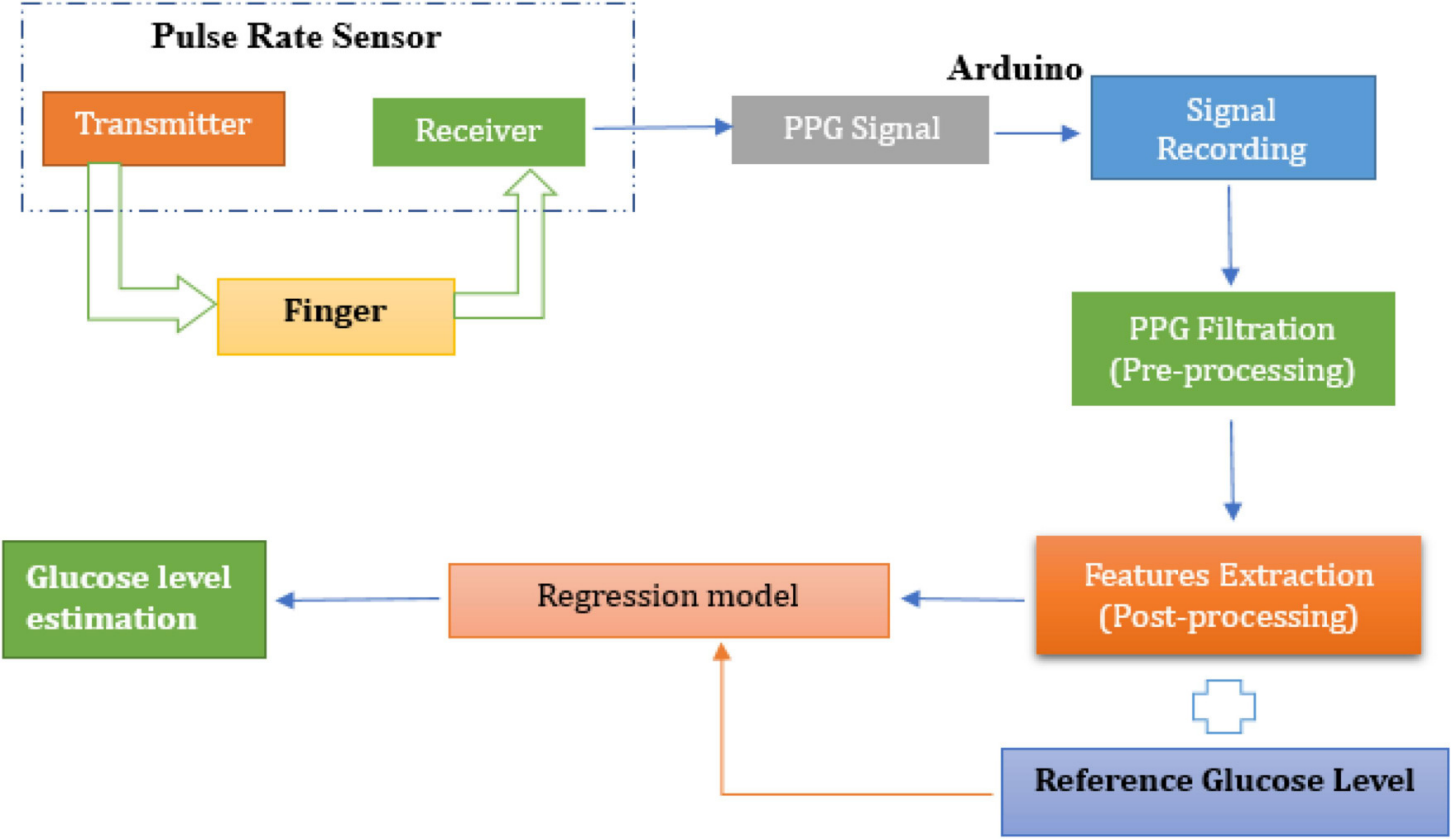
Proposed working mechanism for non-invasive glucose monitoring using pulse sensor.

The methodology involved numerous interconnected steps, ranging from signal acquisition to the development and evaluation of regression models, with the goal of developing a non-invasive glucose monitoring tool based on PPG signals. A visual representation of proposed working mechanism is illustrated in Figure 1.

This study employed a non-invasive approach for blood glucose estimation using PPG signals collected from a fingertip pulse sensor. The sensor, utilizing a green LED light source, detected blood volumetric changes in the fingertip, and the resulting raw PPG signals were recorded using microcontroller programmed through the Arduino Integrated Development Environment (IDE, version 2.0.4, Arduino AG, Switzerland). To enhance signal quality, digital noise filtering techniques were applied before feature extraction. Following pre-processing, key temporal and spectral features were extracted using MATLAB (R2023a, The MathWorks, Inc., Natick, MA, USA), including peak amplitudes, pulse rate, and frequency-domain metrics. The dataset was then transferred to Python (version 3.10, Google Colab environment, Google LLC, Mountain View, CA, USA) for further feature engineering, refining the most informative features for glucose estimation. Various regression models including Random Forest, Support Vector Machine, K-Nearest Neighbor, XGBoost, and Decision Tree were trained and evaluated to determine their effectiveness in predicting glucose levels. The performance of these models was assessed using standard evaluation metrics, with reference glucose values obtained via glucometer serving as ground truth. The following sections provide a detailed breakdown of each methodological step.

### 3.1 Illumination source and sensor selection

**Figure 2:**
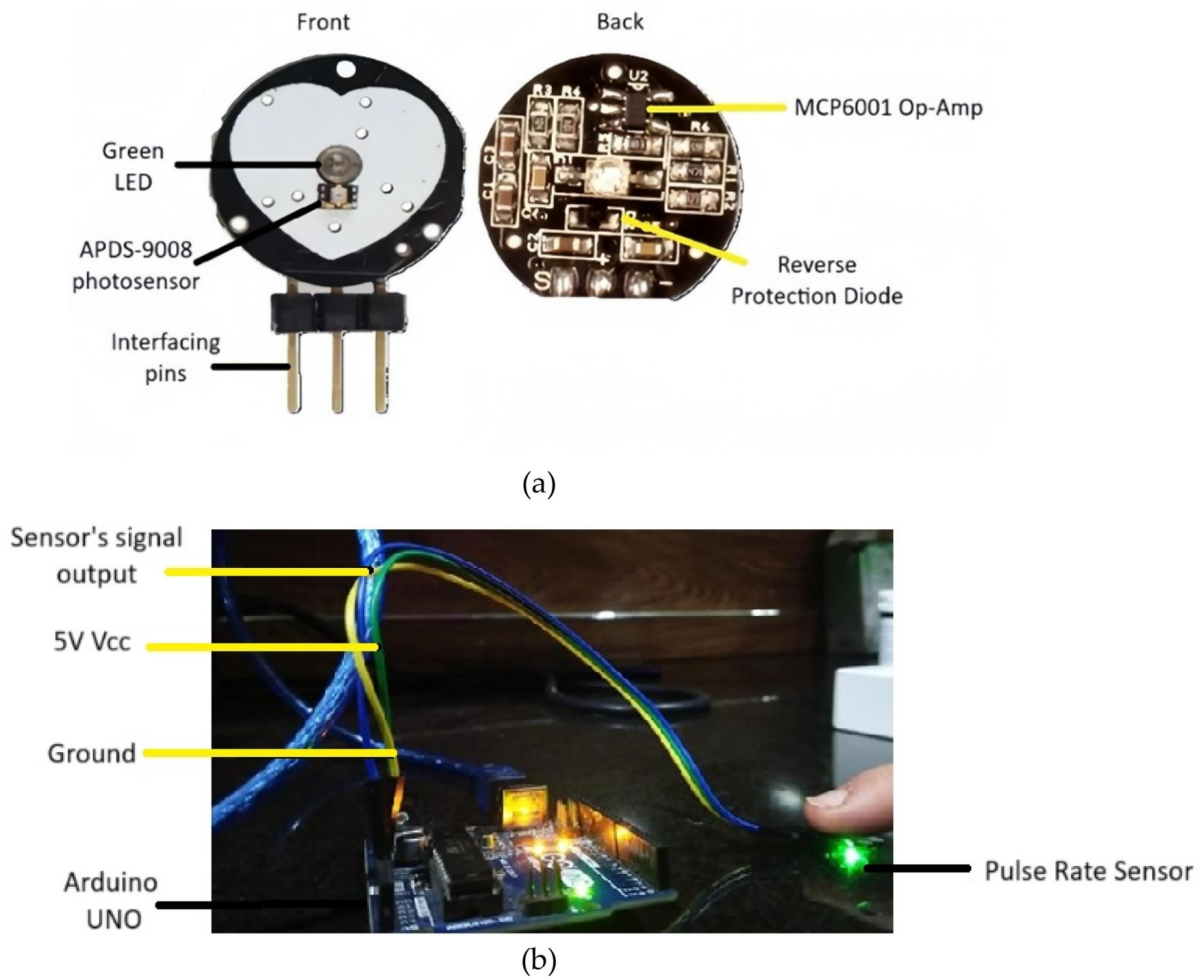
**(a)** Key components of pulse rate sensor **(b)** PPG acquisition setup involving pulse rate sensor interfaced with arduino Uno.

Green light was chosen for PPG signal acquisition because it can effectively penetrate the skin and reach the dermis layer. The dermis contains vital information about blood glucose levels due to its rich network of blood vessels (34). The developed system employs a Pulse Sensor SEN-11574, sourced locally from electronic suppliers in Pakistan and is originally manufactured by SparkFun Electronics (Niwot, Colorado, USA). The sensor integrates a high-intensity green LED (565 nm) and a high-sensitivity photodetector optimized for 525 nm to measure PPG signals. The key components of the sensor are labelled in Figure 2a. The sensor runs from a 5V power supply while using less than 4 mA of current, which makes it power-efficient. The sensor features an onboard amplification circuit and a bandpass filter (0.5–3.0 Hz) to minimize motion artifacts and ambient light interference, ensuring accurate pulse detection. The specifications of the key components involved in the signal acquisition and processing are summarized in Table 2.

**Table 2:** Overview of hardware components and acquisition setup used for PPG signal collection and processing.

System element	Model/Type	Key specifications
Microcontroller	Arduino Uno R3 (Arduino AG, Italy)	10-bit ADC, USB interface
PPG sensor module	Pulse Sensor SEN-11574 (SparkFun Electronics, USA)	Green LED (565 nm), APDS-9008 photodetector, MCP6001 Op-Amp
Filtering component	Onboard Analog Bandpass	Bandpass range 0.5–3 Hz to suppress motion and ambient noise
Real-time data logging	PLX-DAQ Excel Interface (Parallax Inc., USA)	Serial data transfer from Arduino to laptop via USB
Power supply	USB (5 V, <4 mA)	External power via Arduino USB

The Pulse Rate sensor is interfaced with the Arduino Uno microcontroller (locally sourced from an electronics supplier in Pakistan) via its analog input pin, enabling the acquisition of PPG signals. The sensor is integrated with Arduino through the setup shown in Figure 2b. When the green LED illuminates the skin, pulsatile blood flow-induced changes in blood volume vary the reflected light intensity. A photodetector detects the changes and converts them to an analog signal, which is amplified and filtered by the onboard circuitry of the sensor. The signal is passed to an Arduino Uno microcontroller, where a 10-bit ADC samples and digitizes the signal at an optimal rate. The digitized PPG is transferred using serial communication to MATLAB for filtering and feature extraction. The pulse sensor is suitable for real-time acquisition of the signal, and throughout each session, 15 s continuous PPG signal was acquired. However, this study focused on comparing discrete physiological states rather than monitoring continuous transitions. While commercially marketed as a pulse rate sensor, it is actually a PPG-based system that can record raw optical waveforms. Here, it is employed in a targeted manner for PPG signal acquisition, from which glucose-related vascular characteristics are derived for non-invasive estimation of blood glucose. By using green light with the Pulse Sensor, PPG signals can be non-invasively captured that reflect the blood volume changes in the dermis and their correlation with glucose levels. This eliminates the need for invasive procedures, making glucose level monitoring more comfortable and user-friendly (36, 37).

### 3.2 Experimental setup and data collection

**Figure 3:**
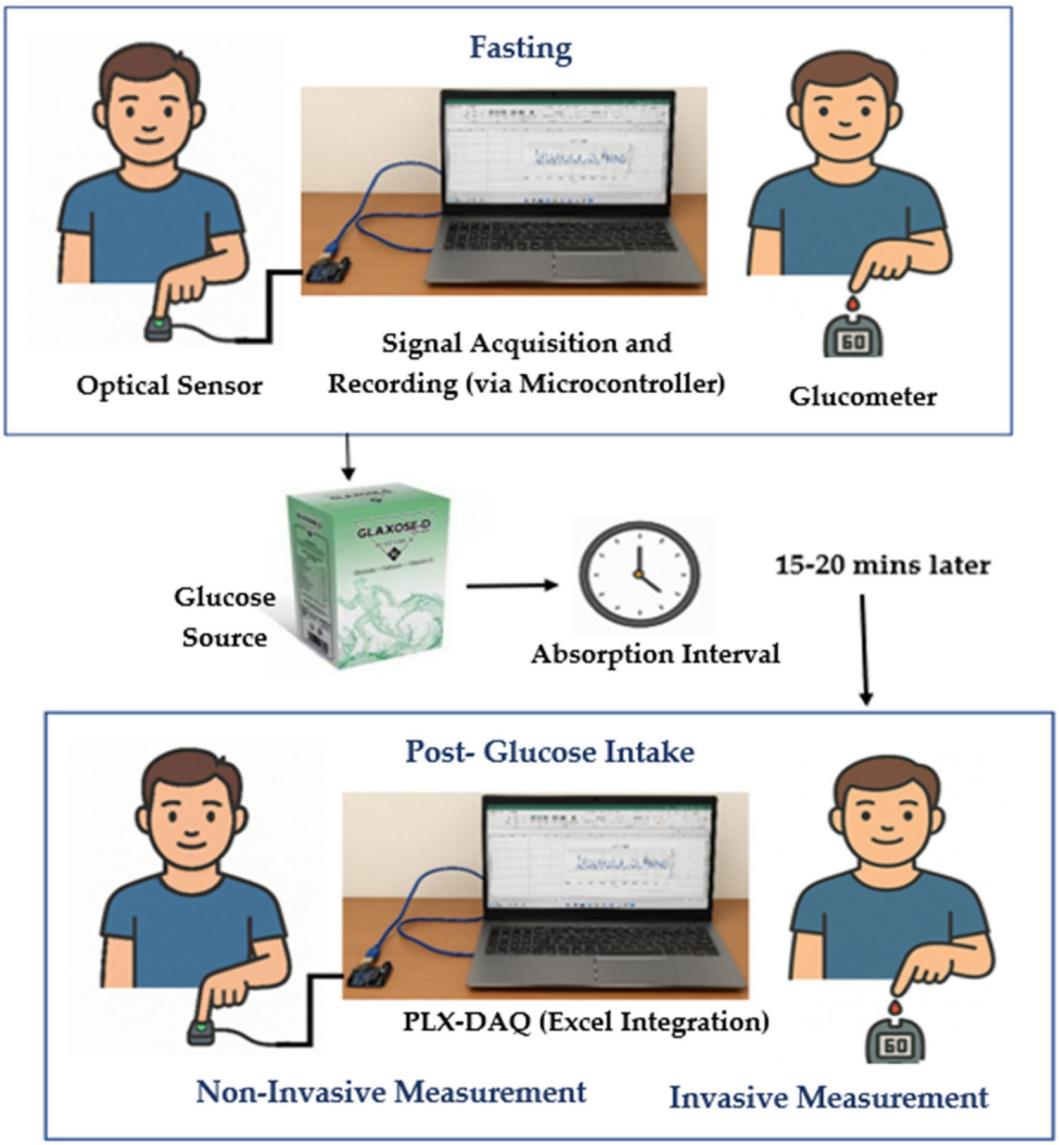
Data collection procedure involving fasting and post-glucose measurements.

This experimental study was conducted over 6–7 months in a university laboratory under controlled conditions. A total of 80 subjects participated, including both young and middle-aged individuals. Young participants were university students, while middle-aged participants included teachers, workers, and other professionals. The study included both male and female subjects, aged 18–50 years. All participants were Pakistani nationals with medium to dark brown skin tones, approximately corresponding to Fitzpatrick types IV–V. The present cohort had less intra-group variability because of relatively uniform pigmentation. Recordings were performed indoors with ambient light minimized, at room temperature (∼22 °C–24 °C). Subjects were seated at rest for 5 min before measurement, and the recorded hand was kept at heart level to maintain consistent blood flow and minimize motion artifacts. As shown in Figure 3, the participant's arm and hand were placed on a stable shelf where the sensor was positioned, further reducing motion during data acquisition.

Before collecting any measurements, all participants were clearly informed about the objectives and protocols of the study. The purpose of the study and the use of the recorded data were explained clearly to ensure transparency. Each participant's voluntary consent was obtained and documented using a written consent form prior to data collection. Each session lasted approximately 40–45 min per participant. All procedures adhered to ethical research standards, ensuring participant safety, privacy, and data confidentiality. The study protocol was reviewed and approved by the institutional ethics committee in accordance with the World Medical Association (WMA) guidelines. The defined inclusion and exclusion criteria are mentioned below with data acquisition setup, shown in Figure 3.

Inclusion criteria:

Healthy individuals aged 18–50 years.
Individuals with stable health conditions, with or without diabetes.

Exclusion criteria:

Subjects with extreme glucose levels outside the typical range, such as a diabetic participant with a glucose level of >200 mg/dL, to avoid skewing the dataset, as only a small number of such cases were available in this university-based participant pool.
Diabetic participants were excluded from post-glucose readings to avoid unsafe blood sugar spikes. Only their fasting readings were recorded, with the study focusing on data variability rather than pre- and post-glucose comparison.

In the first instance, subjects were in a fasting state for approximately 8 h (38). Reference capillary blood glucose readings were obtained using a glucometer via finger prick. Simultaneously, a 15 s PPG signal was recorded non-invasively from the subject's finger using a PPG acquisition setup. In the second instance, the subject was given 15–20 g of Glaxose-D Instant Energy Powder (GlaxoSmithKline Consumer Healthcare, Pakistan) to elevate blood glucose levels. However, this study did not specifically aim to analyze the direct correlation between glucose intake and PPG waveform variations. After 15–20 min of glucose intake (26, 38), the procedure was repeated and PPG signals along with reference glucose measurements were recorded. Diabetic subjects were not included in this phase to prevent unsafe rise in sugar levels. Prior research has shown that glycemic responses to identical glucose intake can differ widely in people who are not diabetic because of variance in insulin sensitivity, absorption in the gut, and other metabolic characteristics (39).

**Figure 4:**
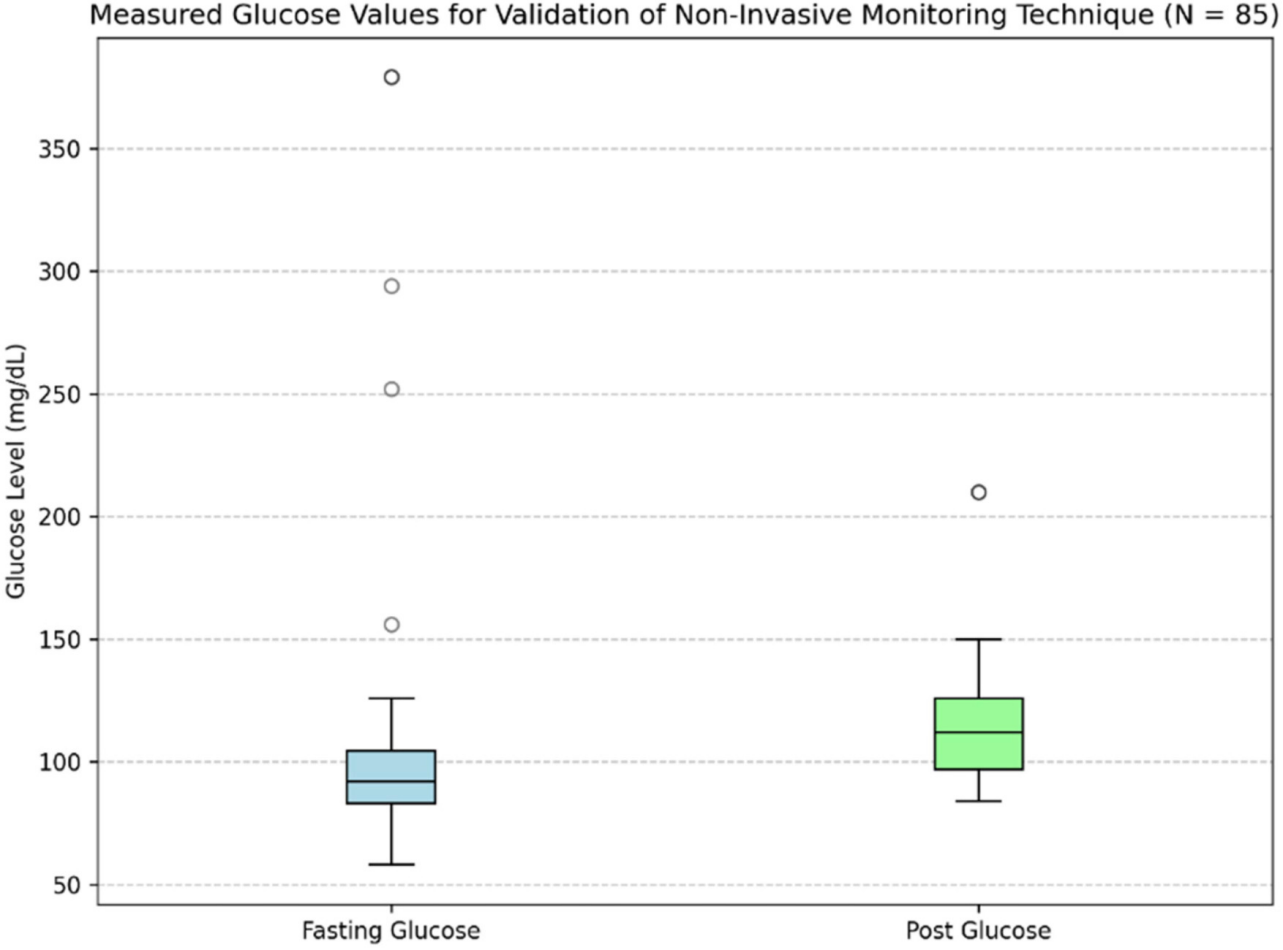
Distribution of fasting and postprandial glucose values used for validation.

PPG signals were recorded from 80 subjects, with each subject undergoing two recording sessions: one in a fasting state and another after glucose intake. In each session, a single PPG profile was obtained using the designed PPG acquisition setup, resulting in two PPG profiles per subject. This approach contributed to a total of 160 PPG profiles. Each PPG recording was paired with a capillary blood glucose reading, resulting in 160 glucose measurements across all subjects. The glucose levels obtained from the glucometer had a mean ± standard deviation of 93 ± 22 mg/dL for the fasting state and 113 ± 30 mg/dL for the postprandial state. The distribution of measurements for both conditions is depicted in the boxplots in Figure 4, and a few fasting values outside the predefined inclusion range were excluded to minimize the influence of outliers on model performance.

Table 3 presents the demographic characteristics of the study participants. After removing outlier glucose readings, this heterogeneous group offered a solid dataset for evaluating PPG signals during fasting and post-prandial conditions. Blood pressure was measured during participant recruitment to monitor general physiological status; however, it was not included in the present analysis, as the focus of this study was limited to PPG-derived features for glucose estimation. Among participants, 10 subjects had pre-existing conditions, such as diabetes with controlled sugar levels or pre-diabetes. All participants involved had glucose levels between 65 and 160 mg/dL, which allowed for variability within a clinically important and safe range as established under study criteria.

**Table 3:** Demographic characteristics of study participants (*n* = 80).

Demographic factor	Details	Number of subjects (*n* = 80)	Percentage (%)
Age group	Young (18–30)	45	56.30
	Middle-aged (31–50)	35	43.80
Gender	Male	42	52.00
	Female	38	47.00
Health Status	Healthy	70	87.50
	Pre-existing conditions	10	12.50
Occupation	Students	44	55.00
	Professionals	36	45.00

Participants with pre-existing conditions were not analyzed separately; all subjects were collectively processed for model development and analysis to capture broader variability in the dataset.

### 3.3 Signal processing

**Figure 5:**
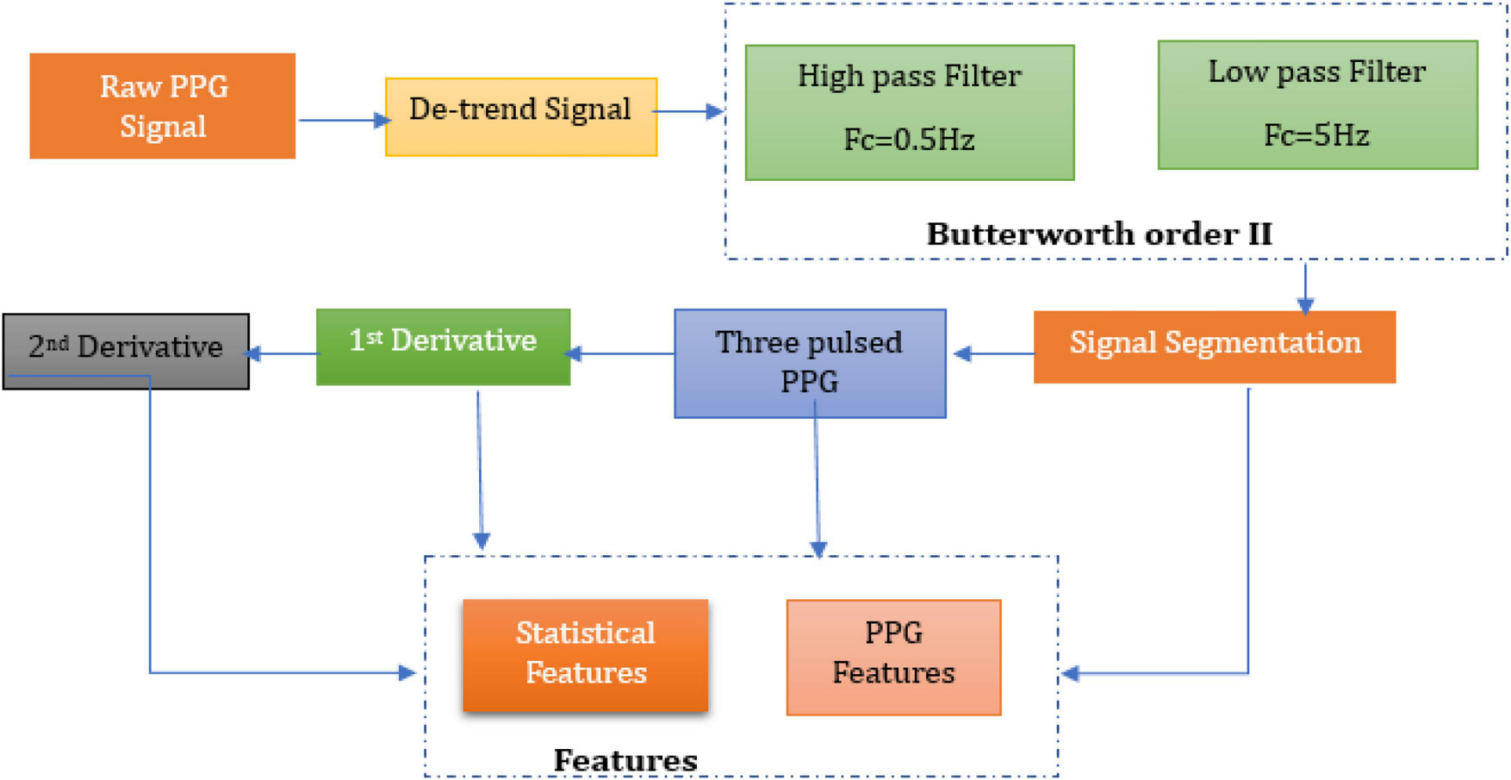
Methodology for pre-processing and post-processing of PPG signals.

The acquired PPG signals are noisy due to motion artifacts, power line noise and other factors involved. These signals need to be processed before feature extraction. Signal processing is conducted in MATLAB (R2023a, The MathWorks, USA), using the Signal Processing Toolbox. Signal preprocessing and feature extraction employed standard functions such as detrend, butter, filtfilt, findpeaks, and diff for derivative-based waveform analysis. It also allows quick implementation of various signal processing algorithms. Flow diagram for PPG signal processing is illustrated in Figure 5, which involves all the steps to be performed for pre-processing and post- processing of PPG signals.

#### 3.3.1 Filtration

For the measured sensor, SNR (Signal-to-Noise Ratio) is crucial because it measures the purity of the PPG signals derived from the green light reflected by the blood. The penetration of green light depends on its wavelength and the light absorption characteristics of tissues; theoretically, greater absorptivity of green light, due to its small wavelength, leads to a better signal-to-noise ratio (37); also, green light's PPG is considered to have the best SNR value (40). However, noise such as baseline drift and motion artifacts interfere with the detection of heartbeats, which are essential features for glucose level prediction. In this regard, previous studies (41–43) have discussed the importance of Butterworth order II filter in short PPG signal filtration, given its smooth frequency response and minimal phase distortion.

**Figure 6:**
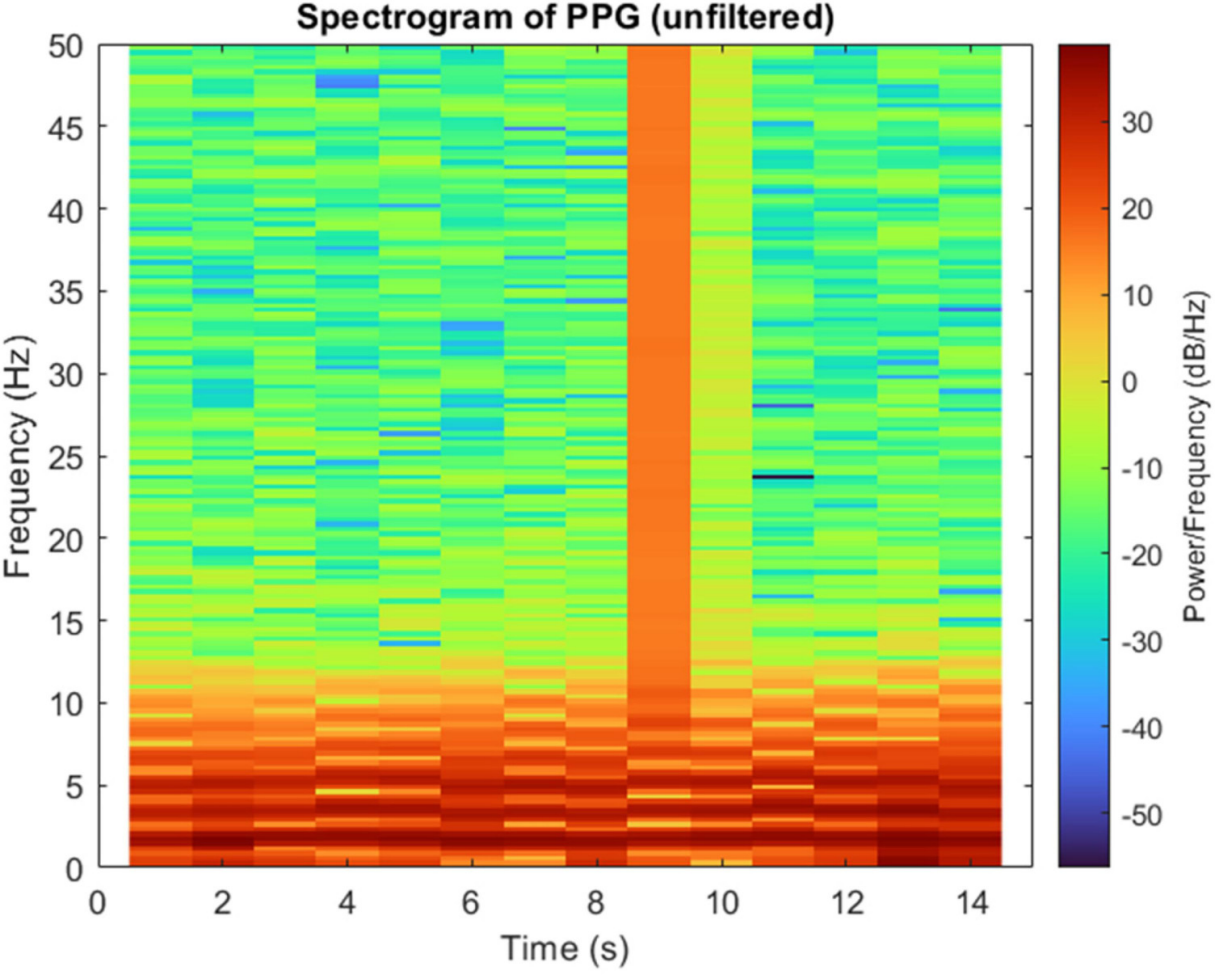
Spectrogram of a PPG signal showing the distribution of power across frequency and time.

In this study, a second-order Butterworth band-pass filter was employed to remove high and low frequency components that could distort the underlying physiological information in the PPG signal. The PPG signals were sampled at approximately 127 Hz, which provides sufficient temporal resolution for accurate waveform analysis and stable filter implementation. The cutoff frequencies were determined based on the spectrogram of the unfiltered signal, as shown in Figure 6. Most of the signal power was observed between 0.5 Hz and 5 Hz. Occasional short, high-energy regions in the spectrogram (e.g., around 9th s) represent transient motion artifacts rather than physiological activity. Therefore, a 0.5–5 Hz passband was selected to retain meaningful cardiac information while attenuating baseline drift and high-frequency noise.

**Figure 7:**
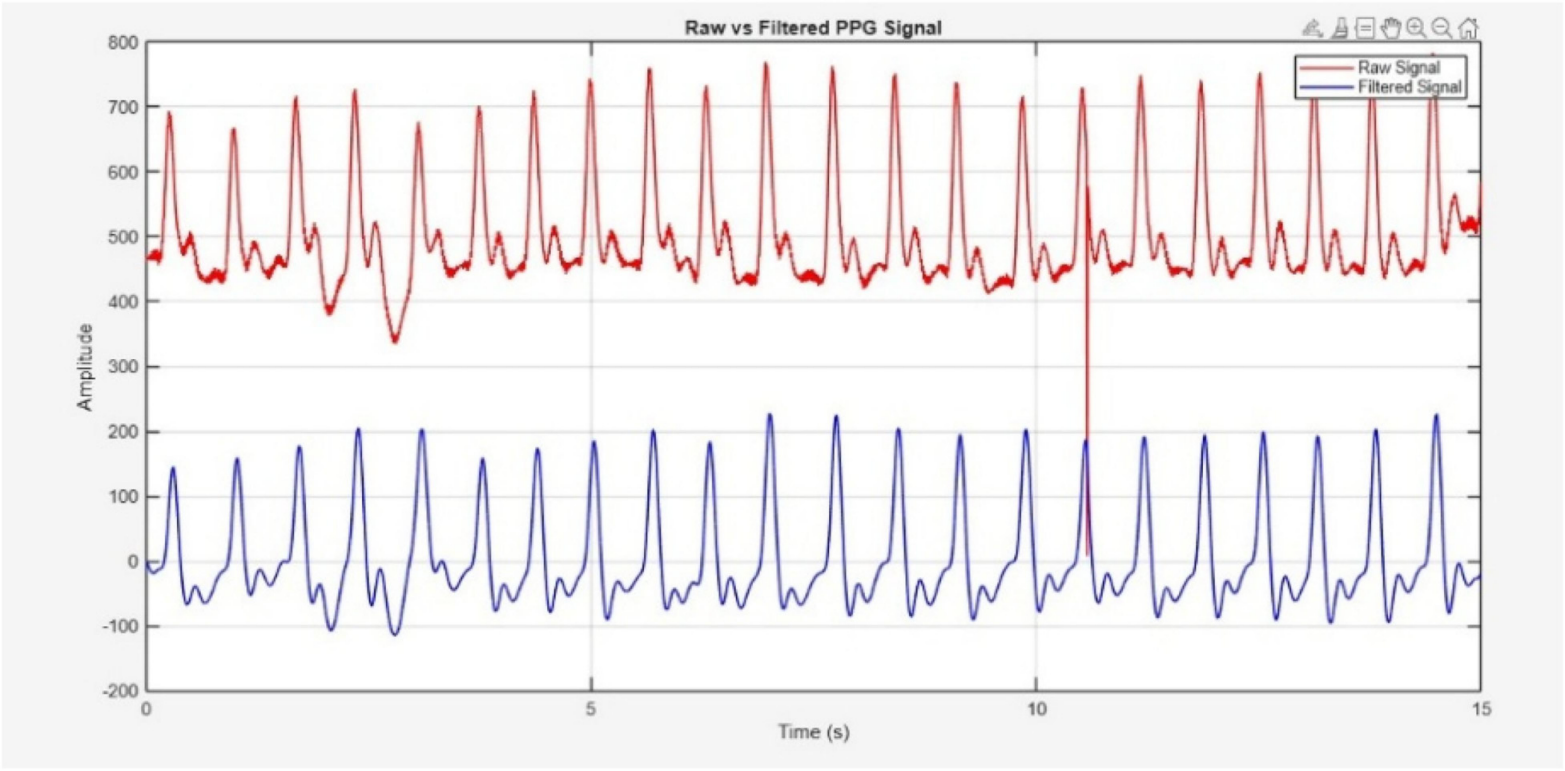
Raw PPG signal acquired through sensor (Red) and filtered signal (blue).

Before filtering, PPG signal was detrended to eliminate the baseline drift and low frequency variations caused by respiration or slight motion. The de-trended signal was then processed using the Butterworth bandpass filter with cutoff frequencies of 0.5 Hz (high-pass) and 5 Hz (low-pass). Visual inspection confirmed that the filtered signal retains the morphological integrity while effectively reducing motion and noise interference. The resulting filtered signal, along with the raw signal, is shown in Figure 7.

SNR for the raw and filtered PPG signals was calculated and compared using a power-based approach. The signal power was defined as the spectral energy within the physiological cardiac band (0.5–5 Hz), while noise power included all remaining spectral components. Unlike harmonic-based definitions (fundamental + first harmonic), the entire physiological band was treated as the signal region. This aligns conceptually with recent PPG studies such as Chen et al. (44), which also compute SNR as a ratio of physiological signal power to noise power. Before filtering, the SNR was 9.37 dB; after filtering, it increased to 20.34 dB, reflecting a 10.97 dB improvement due to effective noise suppression while preserving physiological information.

#### 3.3.2 Segmentation

**Figure 8:**
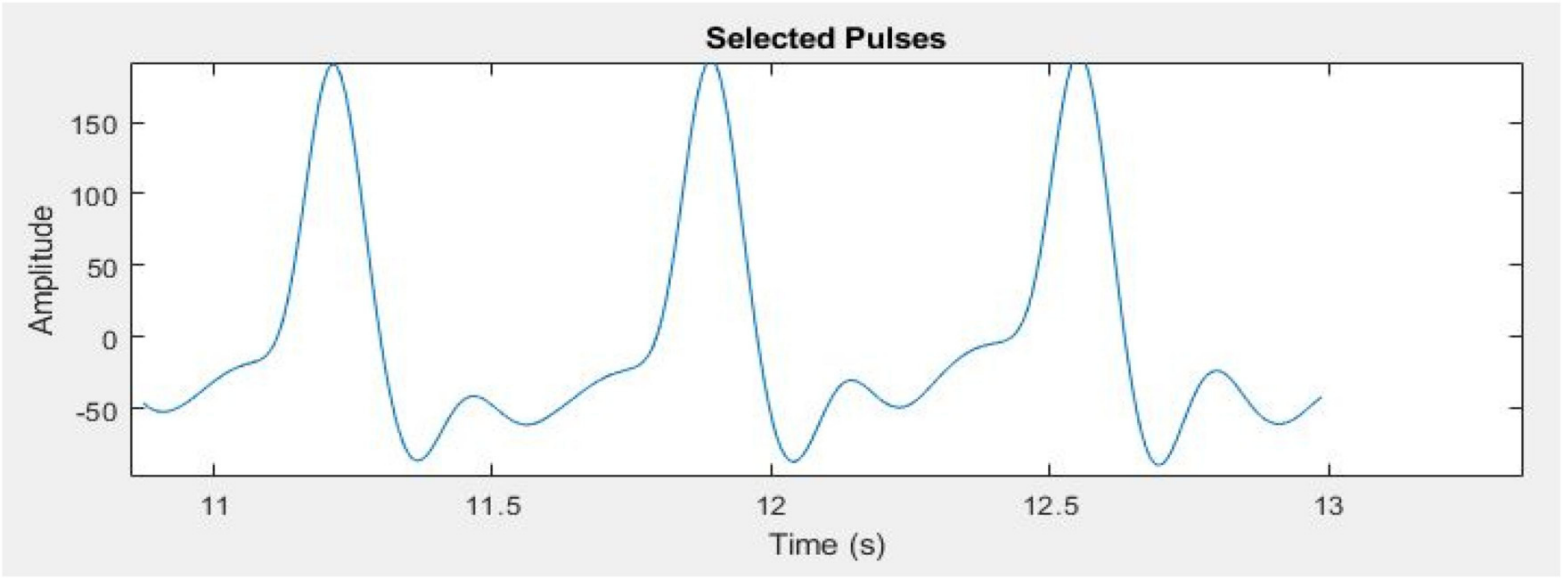
Three segmented pulses from entire PPG signal for further processing.

Signal segmentation is essential in the analysis of PPG signals for the identification and selection of specific pulses of interest. To segment the PPG signal based on its periodicity, an enhanced moving window approach is employed to identify the peak and valley values within the signal (45). The window size was determined using the physiological heart rate range of 60–100 beats per minute, corresponding to one cardiac cycle occurring approximately every 0.6–1.0 s. This range reflects the normal resting HR of healthy adults as reported in clinical literature and supported by the American Heart Association (46). Selecting the window based on this range ensured that each segment encompassed one complete cardiac cycle, allowing accurate identification of systolic peaks and diastolic valleys without signal overlap or false detections. MATLAB was later used to implement the enhanced moving window algorithm. This algorithm continuously adjusts the window along the signal's time axis, detecting peaks and valleys in each timeframe (47). Peaks and valleys correspond to the systolic and diastolic phases of the heart cycle. Based on the observed valley points, the signal was split into regular intervals. These valley spots show the end of one pulse and the start of the next. The signal was segmented in a way that each segment represented a complete PPG pulse. Three consecutive pulses were segmented from the signal, as shown in Figure 8 for detailed analysis, in order to reduce the data size while performing signal processing algorithms.

#### 3.3.3 Features extraction

Statistical as well as peak features from the segmented three pulses are extracted. Features are extracted from the original signal as well as from first and second derivative of PPG signal. The Velocity Photoplethysmogram (VPG) was computed using Equation (1), and the Acceleration Photoplethysmogram (APG) was derived from VPG as shown in Equation (2).

$$\mathrm{VPG}=\frac{d}{\mathrm{dt}}(\;\mathrm{ppg})=\frac{d}{\mathrm{dt}}[y(t+1)-y(t)]$$ (1)

$$\mathrm{APG}=\frac{d}{\mathrm{dt}}(\mathrm{vpg})=\frac{d}{\mathrm{dt}}[y(t+1)+y(t-1)-2y(t)]$$ (2)

**Figure 9:**
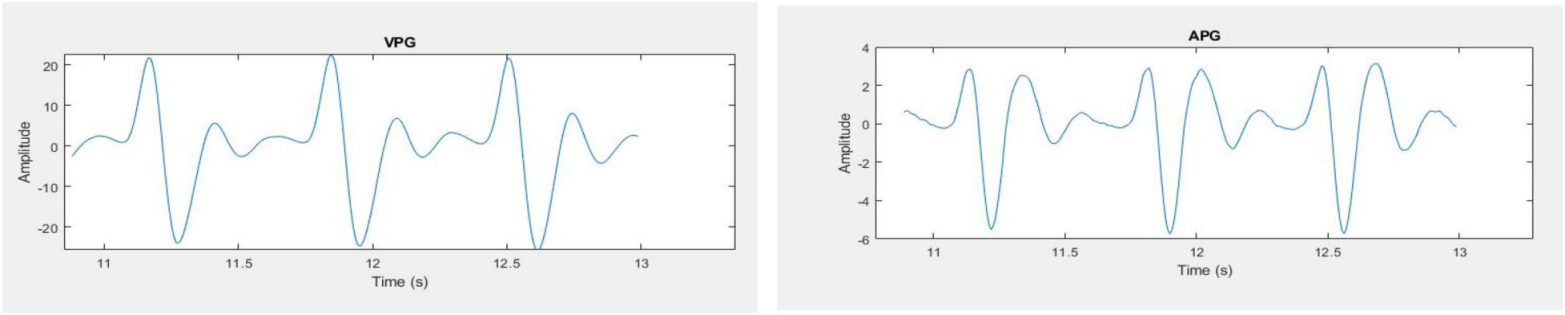
PPG signals (left) first derivative (right) second derivative.

The first derivative and second derivative of the selected pulses are shown in Figure 9. Different ratios associated with the second derivative hold important physiological information. Second derivative is more commonly used than the first derivative. In literature, the second derivative is also called acceleration photoplethysmogram because it is an indicator of acceleration of the blood in the finger (48).

**Figure 10:**
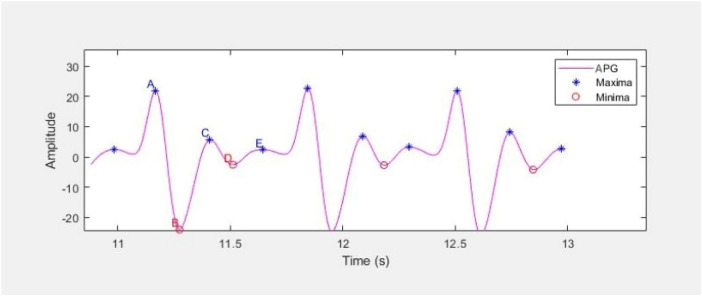
APG waveform representing the a, b, c, d and e parameters as maxima and minima.

APG waveform comprises of four systolic waves (a, b, c and d wave) and one diastolic wave (e wave) as shown in Figure 10. The five a, b, c, d, e parameters as well as their ratios, have been shown to express significant information about cardiovascular condition of the person (48). a wave represents early systolic positive wave, b-wave shows early systolic negative wave, c-wave represents late systolic re-increasing wave, d-wave represents late systolic re-decreasing wave and e-wave is the early diastolic positive wave and represents the dicrotic notch.

The characteristic points a, b, c, d and e are extracted from three consecutive waveforms using MATLAB built-in function findpeaks, and their average is considered for further analysis. All the extracted features are listed in Table 4, and the waveform features follow the standard definitions provided in Elgendi's PPG analysis study (48).

**Table 4:** Features extracted from processed PPG signals.

Statistical features	PPG waveform features	
PPG signal, VPG and APG	Waveform features	APG's ratios
Minimum (Min)	Systolic Peak (S_Peak)	b/a
Maximum (Max)	Diastolic Peak (D_Peak)	c/a
Root Mean Square (RMS)	Inter Peak Interval (P_Pint)	d/a
Signal Energy (S_Energy)	Pulse Width	e/a
Mean	Pulse Rate (P_Rate)	(b-c-d-e)/a
Standard Deviation (SD)	Trough interval - Duration between successive D_Peak (T_interval)	(b-e)/a
Variance (Var)	–	(b-c-d)/a
–	–	(c + d –b)/a

#### 3.3.4 Features preprocessing

Features pre-processing and selection are the most important part of ML to make the data suitable for training. All these steps have been carried out before training the model. Outliers have been identified and removed by visualization through a scatter plot. The extracted features were exported from MATLAB and imported into Python for machine learning model development using a standardized CSV format to ensure synchronization between environments. The following steps are involved in features pre-processing:

##### 3.3.4.1 Features scaling

There was a significant difference between maximum and minimum values of the features; therefore, Min-Max scaling was performed (49). F' indicates the new feature value, whereas F indicates old feature value in Equation (3).

$$\;F^{\prime }=\frac{F-\min}{\max-\min}(\mathrm{ma}x_{\mathrm{new}}-\mathrm{mi}n_{\mathrm{new}})+\min_{\mathrm{new}}$$ (3)

##### 3.3.4.2 Features selection

Out of the extracted features, highly correlated features are shortlisted using Pearson's correlation coefficient (r) approach (50). Depending upon the scores in the heat map, less correlated features are eliminated to avoid irrelevant and redundant features. It helped in reducing the dimensionality of the data. Pearson's correlation coefficient (r) is shown in Equation (4).

$$r=\frac{n\left(\sum \mathrm{ab}\right)-\left(\sum a\right)\left(\sum b\right)}{\sqrt{\left[n\sum a^{2}-{\left(\sum a\right)}^{2}\right]}-\left[n\sum b^{2}-{\left(\sum b\right)}^{2}\right]}$$ (4)

Where *n* = number of pairs of score, ∑a = sum of a scores, ∑b = sum of b scores, ∑a^2^ = sum of squared a scores, ∑b^2^ = sum of squared b scores, ∑ab = sum of the product of paired scores 40.

#### 3.3.5 Features engineering

The highly correlated features were chosen to be engineered to improve their relevance. These included features from the original PPG waveform, its first (VPG) and second (APG) derivatives. Six engineered features were obtained by taking corresponding statistical metrics i.e., maximum (Max), mean, SD, and variance (Var) of PPG, VPG, and APG signals, combined with the systolic-to-diastolic peak ratio and SD-to-mean difference ($SD_{\mathrm{mean}}$). These engineered features are mathematically expressed in Equations (5–10) and employed for training and validating the models for estimating glucose level.

$$\mathrm{Maximum}=\frac{{\mathrm{Max}}_{\mathrm{PPG}}+{\mathrm{Max}}_{\mathrm{VPG}}+{\mathrm{Max}}_{\mathrm{APG}}}{3}$$ (5)

$$\mathrm{Mean}=\frac{{\mathrm{Mean}}_{\mathrm{PPG}}+{\mathrm{Mean}}_{\mathrm{VPG}}+{\mathrm{Mean}}_{\mathrm{APG}}}{3}$$ (6)

$${\mathrm{Peak}}_{\mathrm{Ratios}}=\frac{\mathrm{Systolic}\;\mathrm{Peak}}{\mathrm{Diastolic}\;\mathrm{Peak}}$$ (7)

$$\mathrm{SD}=\frac{{\mathrm{SD}}_{\mathrm{PPG}}+{\mathrm{SD}}_{\mathrm{VPG}}+{\mathrm{SD}}_{\mathrm{APG}}}{3}$$ (8)

$$\mathrm{Var}=\frac{{\mathrm{Var}}_{\mathrm{PPG}}+{\mathrm{Var}}_{\mathrm{VPG}}+{\mathrm{Var}}_{\mathrm{APG}}}{3}$$ (9)

$${\mathrm{SD}}_{\mathrm{mean}}=\mathrm{SD}-\mathrm{Mean}$$ (10)

### 3.4 Regression analysis

Regression analysis was done on a few chosen aspects of the obtained signals and their references in order to estimate the glucose level. In order to assess and compare the effectiveness in non-invasive quantitative estimation of glucose level, various regression models were trained on the signal features that were recovered from the pre-processed signals (26, 51). The models that are being studied are decision trees, XGBoost, K-Nearest Neighbour, Random Forest, and Support Vector Machine. All regression models were implemented in Python using Google Colab (Python 3.8 runtime) with scikit-learn 1.0.2 and XGBoost 1.6.0. Numerous studies have employed ensemble models, such as Random Forest Regression (RFR) and XG Boost, to estimate glucose levels (16, 25, 52). RFR frequently outperforms other regression algorithms in the efficient prediction of biological variables from PPG data, as evidenced by numerous research (53).

In this study, a 50/50 split between training and testing datasets is adopted to ensure both training and testing sets contain a sufficient number of samples for reliable model training and unbiased evaluation. Each of the 80 participants contributed two PPG recordings (fasting and postprandial), yielding a total of 160 signal samples i.e., 80 for training and 80 for testing. Although training proportions above 70% are common in large datasets, such a division can compromise statistical reliability when dealing with small sample counts. With only 160 total samples, a higher training ratio (70/30, 80/20) would have left insufficient test samples to generate statistically meaningful performance metrics and could exaggerate apparent model accuracy. The 50/50 partition therefore provided a balanced approach that preserved both learning capacity and evaluation robustness. Random sampling was applied to ensure representative distribution across subsets and reduce potential bias. A validation set was not used in this study due to dataset size limitations. Without an independent validation subset, hyperparameter tuning and model selection could not be fully optimized, which may slightly affect the generalizability of the results. Nonetheless, the relatively small dataset made an additional split impractical, as it would have reduced statistical power and increased variance in both training and testing. The testing subset was kept fully independent to minimize overfitting and objectively assess model generalization. Future work with larger datasets will incorporate k-fold cross-validation and a dedicated validation subset to enhance robustness and reproducibility.

## 4 Results

**Figure 11:**
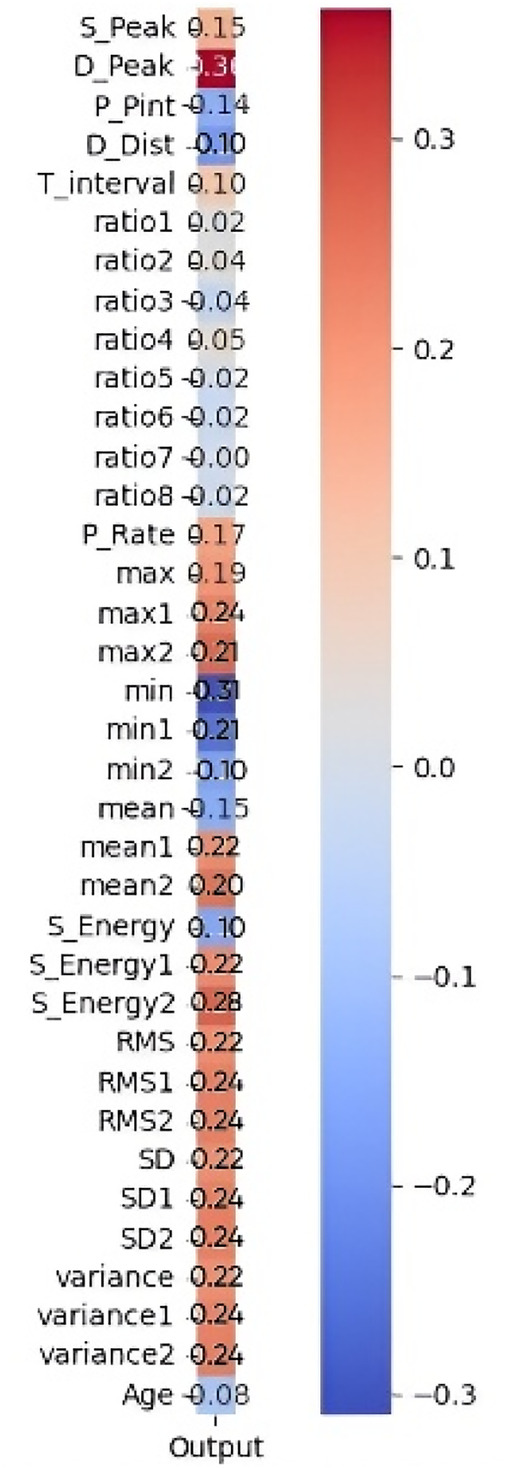
Pearson correlation heatmap of 35 features with the output.

The correlation outcomes between the extracted PPG features and measured blood glucose levels are presented in Figure 11. The heatmap illustrates the strength and direction of these relationships.

Features exhibiting a strong positive correlation are highlighted in red tones, indicating a greater influence on glucose level estimation. In contrast, features with weak or negative correlations (closer to zero) are represented in blue tones and were considered less relevant for prediction. Among the highly correlated features, S_Peak, D_Peak, and Max show strong relationships with glucose levels, suggesting their significance in the predictive model. Conversely, features such as Age, Variance, and Mean2 (mean of APG) display negative correlations, indicating an inverse relationship with glucose levels. The most influential features are prioritized to improve model performance by reducing dimensionality, eliminating noise, and enhancing interpretability. The shortlisted features, as detailed in Table 5, were further processed through feature engineering to enhance their predictive power.

**Table 5:** Shortlisted features after applying Pearson's correlation analysis.

PPG features	Statistical features
Systolic peak	Maximum
Diastolic peak	Mean
Pulse rate	RMS
Troughs interval	Standard deviation
–	Variance
–	Signal energy

**Figure 12:**
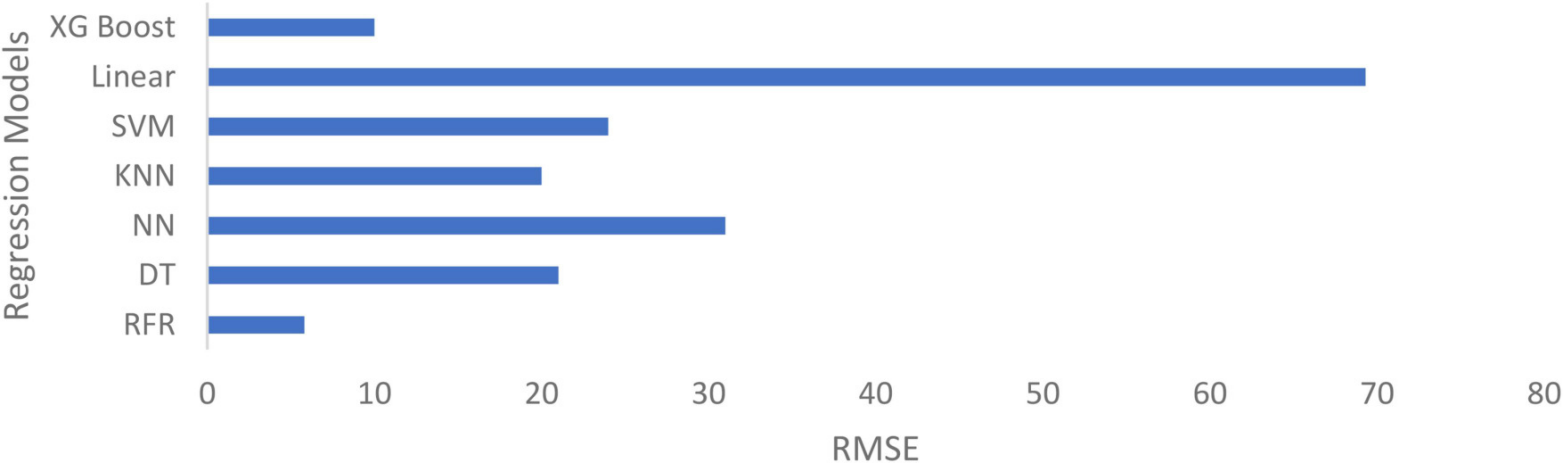
Comparison of performance of different regression models. (XG Boost, extreme gradient boosting; SVM, support vector machine; KNN, K-nearest neighbors; NN, neural network; DT, Decision tree, RFR, random forest regression).

Various regression models were trained, with the RFR model demonstrating the highest predictive performance in glucose level estimation. The RFR model (n_estimators = 100, max_depth = 10, min_samples_split = 2, min_samples_leaf = 1, max_features = “sqrt”, bootstrap = True, random_state = 42) outperformed other models, consistent with previous studies (7, 9, 25), highlighting the efficiency of RFR in glucose prediction. Linear Regression performed poorly due to the dataset's non-linear nature, while XG Boost yielded acceptable results. Figure 12 compares the RMSE (Root Mean Square Error) of different regression models, showing minimal error for the RFR model.

**Figure 13:**
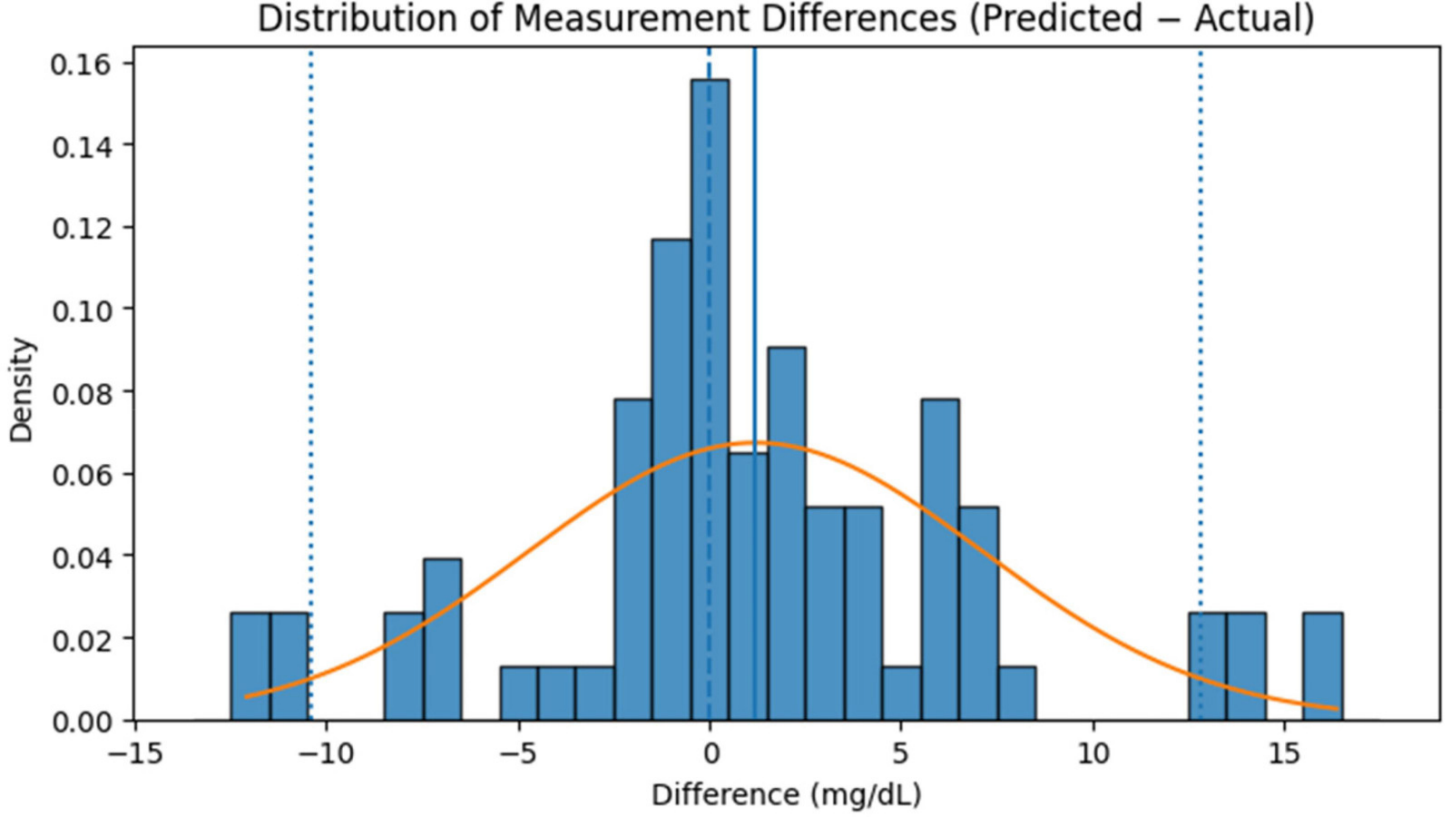
Distribution of measurement differences (predicted−actual) between reference glucose levels and values estimated by the RFR model on the independent test dataset.

Since the RFR model demonstrated the best predictive performance, subsequent analyses (Figure 13 and Table 6) are based on RFR-estimated glucose values. Figure 13 illustrates the distribution of measurement differences (Predicted−Actual) between estimated and reference glucose levels. The histogram shows that most differences are concentrated near zero, indicating minimal systematic bias in the model's predictions. The overlaid red curve represents a fitted normal distribution, confirming that the deviations follow an approximately Gaussian pattern. The solid blue vertical line marks the mean of the differences, positioned close to zero, while the dotted blue lines denote the ±10 mg/dL limits encompassing most observations. While the dataset primarily lies within a moderate glycaemic range (80–135 mg/dL), inter-subject variability was still effectively captured by the model. This consistency demonstrates the robustness of the developed non-invasive model for reliable glucose estimation within moderate glycaemic levels.

**Table 6:** Comparison of reference and predicted glucose levels obtained using the RFR model across different glucose ranges, evaluated on the independent test dataset (*n* = 80).

Glucose level range (mg/dL)	Number of samples (*n*)	Mean predicted (mg/dL)	Mean actual (mg/dL)	Mean difference (mg/dL)	SD predicted (mg/dL)	SD actual (mg/dL)	Mean difference ± SD of differences
<90	24	75.62	80.73	−5.11	5.12	4.84	−5.11 ± 0.80
90–120	30	103.42	107.10	−3.68	9.37	10.03	−3.68 ± 1.24
≥120	26	127.86	133.47	−5.61	5.53	5.56	−5.61 ± 0.75

A key strength of the model is its ability to maintain the predictive performance when tested on subjects not included in the training set. It performed consistently across diverse physiological conditions, including fasting and post-glucose intake states, within a population aged 18–50 years. To further assess the model's performance, a statistical analysis was conducted where glucose values were divided into three ranges: <90 mg/dL, 90–120 mg/dL, and ≥120 mg/dL. The three categories were chosen because there were relatively few points higher than 140 mg/dL or lower than 70 mg/dL in the distribution, such that there would be ample representation within each range. The comparison between PPG-predicted values and reference BGM values for each range is given in Table 6.

The estimated means gradually rise across the glycaemic ranges, corresponding to the actual values and illustrating the model's ability to discriminate between glycaemic states rather than regressing to a central mean. A continuous but minor negative bias is seen, particularly in the lower and upper categories. Importantly, the standard deviations of prediction errors are modest across all ranges (0.75–1.24 mg/dL), showing consistent and steady performance across participants. These findings demonstrate the model's sensitivity to physiological variation in PPG signals and its suitability for non-invasive glucose determination within a clinically relevant range.

**Figure 14:**
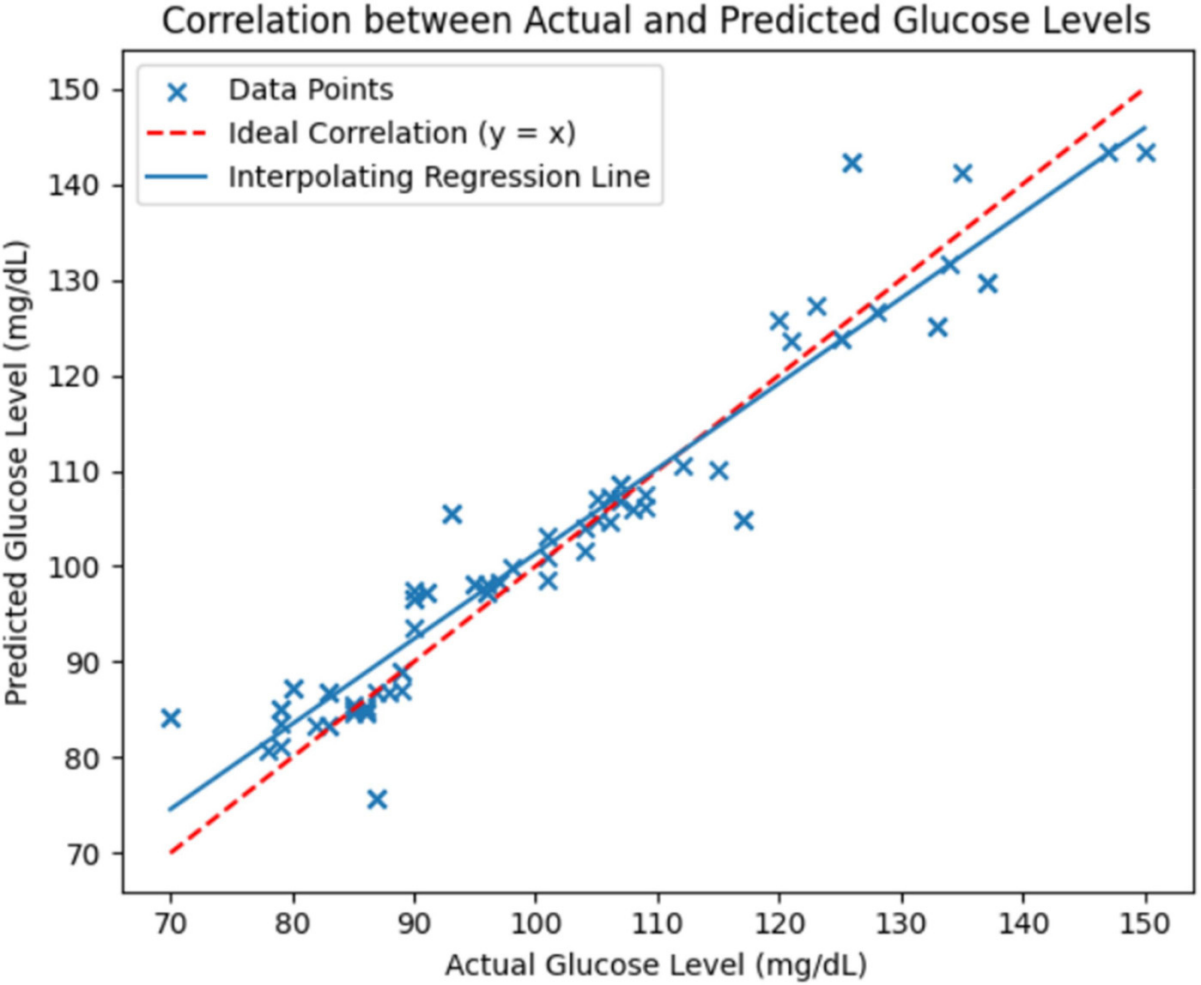
Correlation between actual and predicted glucose levels using the RFR model evaluated on the independent test dataset. The red dashed line represents the ideal correlation (y = x) and the solid blue line shows the interpolating regression trend.

Figure 14 further shows the correlation between actual and predicted glucose levels using the RFR model. The red dashed line indicates the ideal correlation, and an interpolating regression line was included to illustrate the overall linear trend between actual and predicted glucose levels, thereby enhancing the clarity of model performance visualization. The close alignment of data points around these lines demonstrates a strong relationship between predicted and reference glucose values, validating the model's predictive reliability.

**Figure 15:**
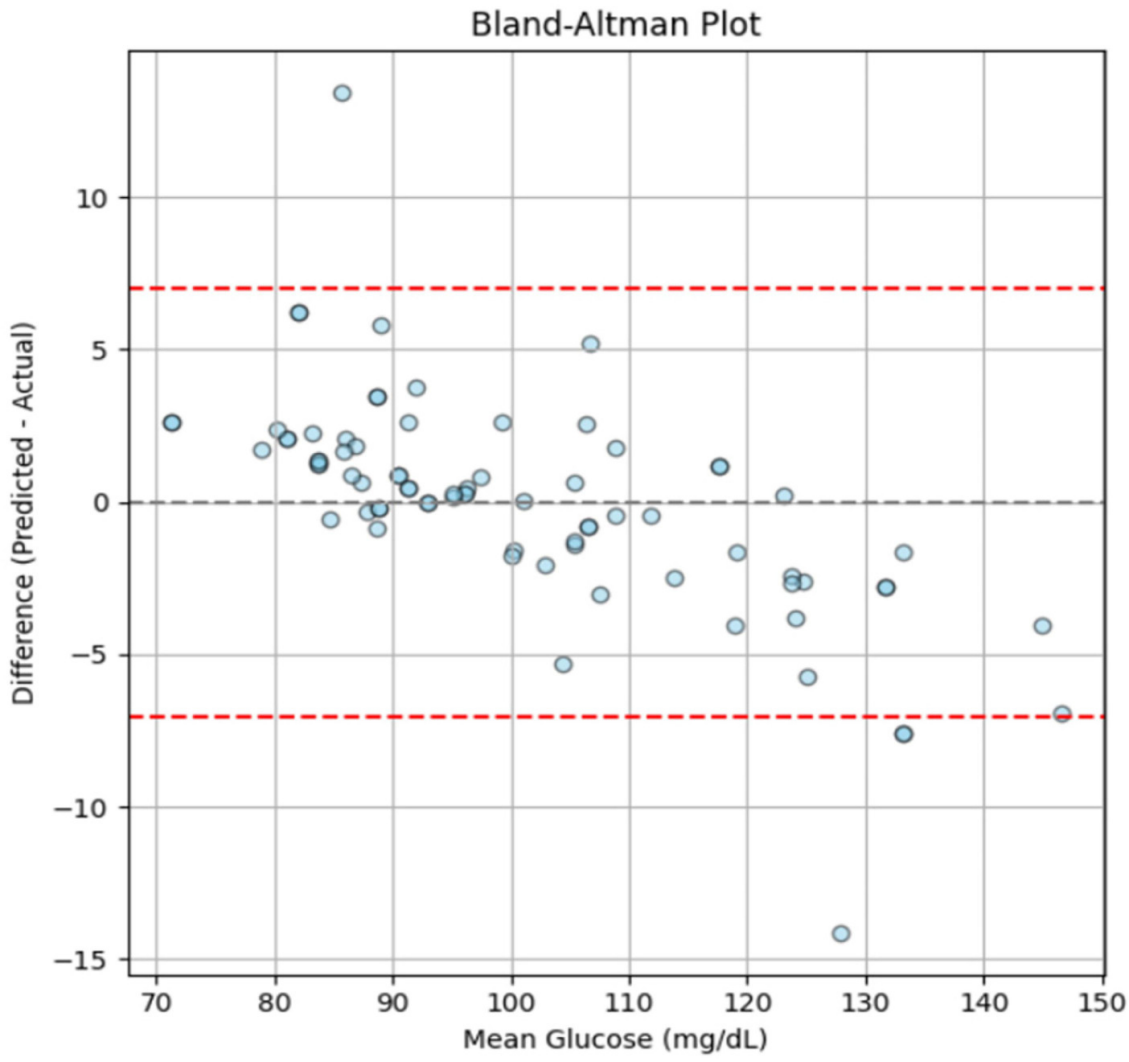
Bland-Altman plot showing the agreement between predicted and actual glucose levels, generated using the independent test dataset.

A Bland-Altman plot was generated in order to further evaluate the degree of alignment between the reference and predicted glucose values as shown in Figure 15.

The findings demonstrate consistent and clinically acceptable predictions across the tested range, with a mean bias approaching zero and almost all data points falling within the 95% range of agreement. However, a slight negative trend can be seen at higher glucose levels (∼120–140 mg/dL), which may result from the smaller number of samples in this range (as shown in Figure 4) and minor underestimation by the model at elevated glucose levels. Nonetheless, the deviations remain within acceptable limits, suggesting that the model maintains consistent predictive performance throughout a range of glucose levels and shows no indication of systematic error.

Overall, the proposed PPG-based glucose estimation model demonstrated superior performance compared to other relevant studies, evidented by metrics of MSE (Mean Square Error), RMSE, MAE, and R^2^, shown in Table 7.

**Table 7:** Performance comparison of the reported work with existing glucose estimation studies, with metrics computed using the independent test dataset only.

Reference	MSE (mg/dL)^2^	RMSE (mg/dL)	MAE (mg/dL)	R^2^
Gupta et al. (9)	N/A	N/A	11.6	0.8
Hina et al. (27)	N/A	12.49	N/A	N/A
Yen et al. (54)	40.7	6.3	5.0	0.99
Nanayakkara et al. (55)	N/A	N/A	N/A	0.58
Fouad et al. (56)	N/A	N/A	N/A	0.91
Guzman et al. (57)	N/A	18.6	16.4	N/A
Zhu et al. (58)	N/A	N/A	N/A	0.93
Reported work	37.2	6.1	4.8	0.92

The results indicate that the proposed method outperformed the existing systems, demonstrating its potential and reliability. These encouraging results form a solid base for further research, supporting the feasibility of non-invasive glucose estimation using PPG signals.

## 5 Discussion

This study evaluated the feasibility of a non-invasive glucose monitoring system using green light PPG signals. The RFR model demonstrated strong predictive performance, with estimated glucose levels aligning well with reference values, particularly within the clinically relevant range of 65 mg/dL to 160 mg/dL. These findings are consistent with prior studies (7, 9, 25) that have validated machine learning models, particularly RF, in glucose estimation. Recently, Alhmiedat et al. (59) also reported a superior performance of RF compared to CNN, as well as other ML models, when applied to cleaned diabetic data sets, with significantly high R^2^ values. Their focus on effective data preprocessing and the strong performance of RF with clinical tabular data aligns with the outcomes of this study, underscoring the reliability and suitability of RF for diabetes prediction tasks.

One of the main strengths of this study is the careful integration of data preprocessing and feature selection techniques, which played a crucial role in improving signal quality and boosting overall performance of the model. Previous studies by Gupta et al. (9) and Anupongongarch et al. (28), struggled with noise and signal instability, limiting predictive performance. In contrast, this study used Pearson's correlation coefficient for feature selection and maintained a controlled data acquisition setup, reducing confounding variables and enhancing model robustness.

Furthermore, unlike single-wavelength NIR spectroscopy (27), which struggles to distinguish glucose from other biomolecules, the proposed PPG-based system demonstrated improved reliability through optimized signal processing. Other approaches, such as those employing polarized light or NIR- LEDs (32, 33), have exhibited sensitivity to skin pigmentation and environmental factors, limiting their applicability. By conducting data collection in a standardized indoor setting and controlling external variables (e.g., ensuring participants' fingers were clean and dry), this study minimized such confounding effects, contributing to the robustness of the acquired results. Another notable aspect of this study was the choice of green light PPG technology, which enhances the practicality of integration into wearable devices. Compared to more complex dual-wavelength or NIR methods (9, 27, 54–58) this system demonstrated superior performance across multiple evaluation metrics, including MSE, RMSE, MAE, and R^2^, reinforcing its potential for non-invasive glucose monitoring.

To induce a rise in blood glucose levels, participants consumed Glaxose-D instead of sugary juices to ensure a standardized glucose dose and avoid variability in sugar content and absorption rates (59). Also, glucose levels were measured only once, 15–20 min post-ingestion, to observe a glucose rise. The American Diabetes Association's (ADA) 15–15 rule supports that blood glucose levels increase measurably within 15 min after consuming 15 g of glucose. Additionally, studies on oral glucose tolerance tests (OGTTs) confirm that plasma glucose levels begin rising within 10–15 min and reach significant levels within 20 min (60). Although more frequent measurements could provide finer insights, repeated finger-pricking was avoided to minimize participant discomfort in a non-clinical setting. CGM was not used in this study due to limited device availability, financial constraints, and the non-insulin-dependent status of the subjects. Capillary blood glucose measurements via glucometer, were selected as a practical and clinically accepted standard, consistent with prior non-invasive glucose monitoring studies (61).

While the study did not explicitly separate fasting and post-glucose levels, measurements were taken from both states to capture natural glucose fluctuations. The use of ensemble learning techniques improved generalization despite a relatively small dataset, mitigating overfitting risks. Additionally, previous research suggests that glucose ingestion may influence PPG waveform characteristics by altering vascular tone and blood flow (62, 63), but this study did not analyze those specific variations. Future research should explore these effects to further refine non-invasive glucose estimation techniques. The consistency of predictions across glycemic ranges verifies the efficacy of signal processing and feature extraction, creating a solid foundation for future applications in real-time PPG analysis and continuous glucose monitoring. Further refinement in signal stability, motion artifact reduction, and implementation in microprocessor-based platforms has the potential for this method to develop into an applicable, non-invasive CGM device.

Despite promising results, this study has several limitations. The sample size was relatively small, with limited representation of diabetic participants, which may restrict the model's generalizability to real-world clinical populations. Extreme variations that are frequently seen in clinical settings were not captured by the measured blood glucose range (65–160 mg/dL). This was mainly due to the limited availability of participants with high glucose levels (>200 mg/dL) and ethical safety considerations in a non-clinical university setting, where administering glucose to diabetic individuals could pose health risks. Including only a few such cases might also have introduced imbalance or outlier effects in an already limited dataset. However, even within a limited clinical range, this inter-individual glycaemic variability significantly altered the PPG properties that were utilised to train the model. Such a variability has been documented in literature and confirms the adequacy of collected dataset for non-invasive glucose modeling (39).

A further limitation is the narrow skin-tone range in our cohort, which may limit generalizability across lighter and darker phenotypes. Moreover, although the dataset included both genders and a range of ages, the study did not conduct a separate analysis to evaluate whether gender or age influenced PPG waveform characteristics. We also did not continuously monitor skin temperature/perfusion or contact pressure, which can influence PPG amplitude and baseline stability. Also, instead of repeated measurements at shorter intervals, glucose levels were measured only once, 15–20 min post-ingestion. While prior studies suggest this timeframe is sufficient to detect a glucose rise, continuous monitoring could have provided deeper insights into glucose dynamics. Additionally, data collection was conducted in a controlled indoor environment, so the system's performance under real-world conditions, including temperature variations, motion artifacts, and stress, remained untested.

In order to better assess the temporal performance and responsiveness of the PPG-based glucose estimation model, future research should combine both CGM and BGM to allow for simultaneous continuous and point-based reference measurements. Future studies will also incorporate Clarke or Parkes error grid analysis to provide a clinically standardized evaluation of glucose estimation accuracy. Moreover, dataset will be enhanced by including a larger and more diverse participant pool covering wider age groups, health conditions, and glucose ranges. This will help capture both the dynamic trends and precise reference values. To validate performance at clinically relevant extremes, the glucose range must be expanded to 400 mg/dl. Furthermore, collecting data during spontaneous glycaemic fluctuations without regulating glucose intake can more accurately represent actual circumstances. This can be facilitated by inviting diabetic participants to manage their glucose levels during longer observation periods, enabling dynamic monitoring without external manipulation. Such meticulously planned procedures would more efficiently test and confirm the durability of PPG-based glucose estimate models, even with fewer people. In addition, future studies should also stratify participants by skin tone, temperature, motion, and evaluate multi-wavelength PPG approaches to mitigate melanin-related optical biases and enhance generalizability. These considerations align with current regulatory frameworks for pulse oximeter devices (64), which form the basis for validating optical wearable technologies and emphasize the need for population-specific performance evaluation.

## 6 Conclusion

The proposed research focused on exploring the potential of green light for estimating blood glucose level in a non-invasive manner. Glucose level has been estimated by applying various regression ML algorithms ultimately achieving the highest R^2^ value (0.92) with RFR model. A custom-made PPG acquisition setup and advanced feature extraction algorithms set this work apart, showcasing the practicality of an accessible alternative to traditional glucometers. The use of green light, which is widely available and less affected by environmental factors, makes it feasible to explore the application of this technology in wearable, real-life devices. This balance of practicality and performance highlights the novelty of the proposed approach. By providing a cost-effective solution, this research paves the way for significantly enhancing diabetic care and accessibility. Future work will extend from this study to optimise algorithms and include more diverse sample sets to increase model performance and applicability, with the aim to support the innovation in CGM technologies.

## Data Availability

The original contributions presented in the study are included in the article/Supplementary Material, further inquiries can be directed to the corresponding author.
